# *Rede Cegonha*: maternal characteristics and perinatal outcomes related to prenatal consultations at intermediate risk*

**DOI:** 10.1590/1980-220X-REEUSP-2021-0248

**Published:** 2022-01-31

**Authors:** Franciele Aline Machado de Brito, Marcia Moroskoski, Bianca Machado Cruz Shibawaka, Rosana Rosseto de Oliveira, Beatriz Rosana de Oliveira Gonçalves Toso, Ieda Harumi Higarashi

**Affiliations:** 1 Universidade Estadual de Maringá, Programa de Pós-Graduação em Enfermagem, Maringá, PR, Brazil.; 2 Universidade Estadual do Oeste do Paraná, Programa de Mestrado Biociências e Saúde, Cascavel, PR, Brazil.

**Keywords:** Prenatal Care, Maternal-Child Health Services, Infant Mortality, Atención Prenatal, Servicios de Salud Materno-Infantil, Mortalidad Infantil, Cuidado Pré-Natal, Serviços de Saúde Materno-Infantil, Mortalidade Infantil

## Abstract

**Objective:**

to analyze the correlation between maternal characteristics and perinatal outcomes, with the number of prenatal consultations performed.

**Method:**

a cross-sectional study, carried out with 1,219 mothers and newborns stratified as intermediate risk according to the *Programa Rede Mãe Paranaense*, adaptation of the *Rede Cegonha* at the state level. Data were collected from the Birth Certificates. Spearman, Wilcoxon and Kruskal-Wallis tests were used to analyze the correlation between the variables of interest.

**Results:**

married women, with higher education, white and aged 30 years or older were the ones who most attended prenatal consultations. With regard to perinatal outcomes, children whose mothers had more frequent prenatal consultations had better Apgar and birth weight scores. High rates of cesarean delivery were identified before the onset of labor.

**Conclusion:**

maternal characteristics influence the process of adherence to prenatal care, impacting perinatal outcomes, indicating the relevance of these risk factors and the need to improve actions aimed at greater compliance with risk stratification and qualified and resolute care for pregnant women at intermediate risk.

## RESUMO

**Objetivo:**

analisar a correlação entre as características maternas e os desfechos perinatais, com o número de consultas pré-natais realizadas.

**Método:**

estudo transversal, realizado com 1.219 mães e recém-nascidos estratificados como risco intermediário de acordo com o Programa Rede Mãe Paranaense, adaptação da Rede Cegonha no âmbito estadual. Os dados foram coletados das Declarações de Nascidos Vivos. Foram utilizados os testes de Spearman, Wilcoxon e Kruskal-Wallis, para analisar a correlação entre as variáveis de interesse.

**Resultados:**

mulheres casadas, com maior escolaridade, da cor branca e com 30 anos ou mais foram as que mais compareceram às consultas de pré-natal. No tocante aos desfechos perinatais, crianças cujas mães tiveram maior frequência nas consultas de pré-natal, apresentaram melhores índices de Apgar e de peso ao nascer. Identificaram-se elevadas taxas de cesariana antes do início do trabalho de parto ocorrer.

**Conclusão:**

características maternas influenciam no processo de adesão ao pré-natal, impactando nos desfechos perinatais, indicando a pertinência destes fatores de risco e a necessidade de aprimorar ações voltadas à maior observância da estratificação de risco e ao atendimento qualificado e resolutivo das gestantes pertencentes ao risco intermediário.

## RESUMEN

**Objetivo:**

analizar la correlación entre las características maternas y los resultados perinatales, con el número de consultas prenatales realizadas.

**Método:**

estudio transversal, realizado con 1.219 madres y recién nacidos estratificados como riesgo intermedio según el *Programa Rede Mãe Paranaense*, adaptado de la *Rede Cegonha* a nivel estatal. Los datos se obtuvieron de los certificados de nacimiento. Se utilizaron las pruebas de Spearman, Wilcoxon y Kruskal-Wallis para analizar la correlación entre las variables de interés.

**Resultados:**

las mujeres casadas, con estudios superiores, blancas y de 30 años o más fueron las que más acudieron a las consultas prenatales. En cuanto a los resultados perinatales, los niños cuyas madres tuvieron consultas prenatales más frecuentes tuvieron mejores puntajes de Apgar y peso al nacer. Se identificaron altas tasas de parto por cesárea antes del inicio del trabajo de parto.

**Conclusión:**

las características maternas influyen en el proceso de adherencia a la atención prenatal, impactando los resultados perinatales, indicando la relevancia de estos factores de riesgo y la necesidad de mejorar las acciones encaminadas a un mayor cumplimiento de la estratificación del riesgo y atención calificada y decidida a las gestantes en riesgo intermedio.

## INTRODUCTION

Infant mortality remains a serious global public health problem, thus pointing to some weaknesses in the public health system^(1)^. Sometimes, such weaknesses also involve economic and sociodemographic factors, which makes their reduction complex and dependent on other areas, especially in developing countries where the articulation of services is normally fragile^(1–2)^.

Although there has been a reduction in infant-juvenile mortality in the last thirty years, as a result of the efforts of managers, health professionals and national and international organizations^(3)^, children continue to face disparities in the chances of survival in the world^(1)^. In 2019, 1.5 million babies under one year of age died globally. In the same year, 14,000 children under the age of five died every day, and 6,700 were newborns, i.e., almost half of all deaths occurred in the neonatal period^(4)^.

It is known that actions aimed at improving the quality of prenatal care, delivery and puerperium directly impact in infant mortality reduction, especially when they emphasize prenatal care. This modality of care is responsible for ensuring continuous care, preventing obstetric problems, reducing the occurrence of prematurity and low birth weight, ensuring pregnant women’s access to health services and healthy deliveries and births^(2)^.

However, even in view of the recognition of its relevance in the context of public health, the healthcare reality has shown that many women do not attend prenatal consultations, a behavior that is repeated in child health monitoring. The reasons for this to occur are variable and range from the social determination of the health-disease process to individual, social and institutional vulnerability^(5)^.

Faced with this scenario of inequities in access and problem-solving capacity in primary care, added to the stagnant infant mortality rate of two-tenths in the last two decades, the Brazilian federal government launched the *Rede Cegonha* (RC – Stork Network) in 2011, which in the state of Paraná was implemented under the name *Programa Rede Mãe Paranaense* (PRMP – Paraná Mother Network Program), becoming the main guideline for maternal and child health services in the state^(6)^.

The PRMP presents as a differential, the risk stratification based on analysis of epidemiological data of its territory, with the addition of intermediate risk. Scientific evidence already indicates that maternal characteristics influence the process of adherence to prenatal care, with impacts on the perinatal outcomes of newborns, as well as sociodemographic factors interfere in women’s prenatal care. In this sense, this study advances the systematic and continuous assessment of maternal and child health services, with a special focus on prenatal consultations in the context of intermediate risk.

Thus, maternal characteristics linked to sociodemographic factors make up the defining criteria of the intermediate risk population of the PRMP. According to the state guideline, this portion of pregnant women and children must be assisted in the network’s specialized clinics by trained professionals^(6)^. However, there is evidence that maternal and child care services are not developing the actions proposed by the program in its entirety. Studies have shown flaws in risk stratification, and the lack of training of professionals working in the network, which implies difficulties in following the protocols, factors that can contribute to the increase in infant mortality rates^(7–8)^.

Therefore, it is essential to carry out assessment studies of maternal and child health services, aimed at the intermediary risk of the *Rede Mãe Paranaense*, in order to verify the relevance of these factors as determinants of health risks for this population. The study is also justified considering the fact that it is a subject still little explored in research in the area of women’s and children’s health. Therefore, the study aimed to analyze the correlation between maternal characteristics and perinatal outcomes, with the number of prenatal consultations performed, within the intermediate risk of the PRMP.

## METHOD

### Design of Study

This is a cross-sectional, analytical study with retrospective data collection. The recommendations of the Strengthening the Reporting of Observational Studies in Epidemiology (STROBE)^(9)^ were used for the study construction and presentation.

### Population

In 2019, 9,176 births were registered in the city, and of these, 5,119 were of children born and residing in the city. From this portion, 1,219 were selected for the study, as they fit into the intermediate risk stratification, according to the criteria of the *Rede Mãe Paranaense* ^(6)^. In cases of multiple pregnancy (5.09%), the mother was considered more than once, as the study’s observation units are the newborns.

### Local

The study site was the city of Maringá, which is located in the state of Paraná, in southern Brazil. The estimated population of the municipality in 2020 was 430,157 inhabitants and the Human Development Index in 2017 was 0.778^(10)^.

### Selection Criteria

Children who meet at least one of the following criteria are stratified as intermediate risk in the state of Paraná: newborns born to black and/or indigenous mothers; children of mothers under 15 or over 40; children of illiterate mothers or with less than three years of education; children of mothers with a history of death in a previous pregnancy (abortion, stillbirth or death); children of mothers under 20 years old; more than three births. Thus, children who presented one or more of these characteristics, classified in the Certificate of Live Birth (CLB) as belonging to intermediate risk, were considered eligible for the study. Residence in another municipality was established as the sole exclusion criterion, even if the birth took place in Maringá.

### Data Collection

The database was made available by the Municipal Health Department in February 2020. The information comes from the CLB for 2019. The choice of this period aimed at analyzing the most recent data available. To obtain the variables of interest, filters were selected in the Microsoft Office Excel program, in order to exclude data from births that did not fit the criteria of interest.

### Data Analysis and Treatment

At first, a descriptive analysis was carried out, in order to characterize the research participants. To describe the results related to maternal characteristics, the absolute and relative frequency were used for the categorical variables, namely: mother’s marital status (married; stable union; single; divorced; widowed; ignored); education years (none; 1 to 3 years; 4 to 7 years; 8 to 11 years; 12 years or more and ignored); mother’s color (white; black; brown; yellow; indigenous and ignored); pregnancy type (single; double; triple or more; ignored). As for numerical variables, simple arithmetic mean, standard deviation and median were used, and these variables were: mother’s age; number of previous pregnancies; number of cesarean deliveries; number of normal births; number of living children; number of dead children; month of pregnancy in which prenatal care started.

With regard to perinatal outcomes, the categorical variables related to prenatal care and delivery were: place of birth (hospital; home; other health facilities); professional who assisted the birth (doctor; nurse/midwife); delivery type (cesarean; normal); induced labor (no; yes and ignored); Caesarean section before the onset of labor (no; yes; not applicable and ignored); Newborn presentation type (cephalic; pelvic or foot; transverse; ignored); method used to estimate gestational age (physical examination; other method is ignored); sex (male or female); anomaly identified (no or yes). Regarding numerical variables, the following were considered: Apgar at the 1^st^ minute; Apgar at the 5^th^ minute; Robson group code; number of weeks of gestation; and birth weight.

To analyze the correlation between the number of prenatal visits and maternal characteristics and perinatal outcomes, Spearman’s correlation test was used for numerical variables and the Wilcoxon and Kruskal-Wallis tests for categorical variables. All analyzes were performed using the statistical environment R (R Development Core Team), version 3.6.2.

### Ethical Aspects

The research project was approved by the Standing Research Ethics Committee of the *Universidade Estadual de Maringá*/PR, under Opinion 3.766.436/2019.

## RESULTS

**Figure 1.**
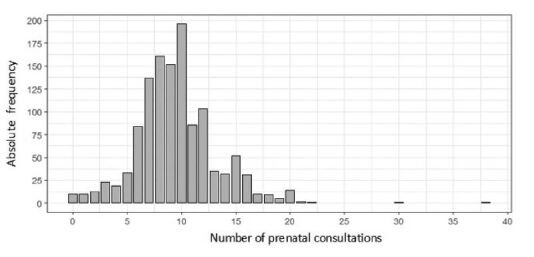
Distribution of the number of prenatal consultations performed by mothers of children stratified as intermediate risk according to the Mãe Paranaense Network – Maringá, Paraná, Brazil, 2019.

Source: Municipal Health Department of Maringá, Paraná, Brazil, 2019.

It is observed in Figure 1 that most women had between seven and 10 consultations (52.99%), and that five pregnant women had more than 20 consultations (0.41%), with a maximum of 38. Highlights it is also noted that 10 (0.82%) did not attend any prenatal consultation. The average number observed was 9.53 prenatal consultations, with a standard deviation of 3.69 and a median of nine, indicating that half of the mothers had at least nine consultations.

Regarding maternal characteristics, 78.18% of the women were married or in a stable relationship. These two groups, together with widows, have the highest average number of prenatal consultations, equal to or greater than nine. The distribution of the number of consultations showed significant differences according to mothers’ marital status (p < 0.001) (Table 1).

**Table 1.** Number of prenatal visits performed by mothers of children stratified as intermediate risk according to marital status, education, mother’s color and pregnancy type – Maringá, Paraná, Brazil, 2019.

Variable	Frequency		Prenatal consultations			p*
	n	%	Mean	Standard deviation	Median	
Mother’s marital status						<0.001^†^
Married	702	57.59%	10.09	3.74	10	
Cohabitation	251	20.59%	9.24	3.40	9	
Single	239	19.61%	8.33	3.59	8	
Divorced	20	1.64%	8.40	1.93	9	
Widow	3	0.25%	9.67	1.53	10	
Ignored	4	0.33%	5.75	3.86	7.5	
Education years						<0.001^†^
None	5	0.41%	7.40	2.07	7	
1 to 3 years	4	0.33%	8.75	3.30	9	
4 to 7 years	60	4.92%	8.62	3.81	9	
8 to 11 years	681	55.87%	9.15	3.61	9	
12 years and older	460	37.74%	10.30	3.64	10	
Ignored	9	0.74%	6.11	4.48	7	
Mother’s color type						<0.001^†^
White	703	57.67%	9.93	3.72	10	
Black	244	20.02%	8.52	3.46	8	
Brown	251	20.59%	9.39	3.58	9	
Yellow	12	0.98%	10.83	2.62	11	
Indigenous	2	0.16%	9.50	9.19	9.5	
Ignored	7	0.57%	6.71	4.89	7	
Pregnancy type						0.001^†^
Single	1156	94.83%	9.43	3.61	9	
Double	59	4.84%	11.07	4.57	10	
Triple or more	3	0.25%	16.00	0.00	16	
Ignored	1	0.08%	7.00	–	7	

Source: Municipal Health Office of Maringá, Paraná, Brazil, 2019.

* p value calculated from Kruskal-Wallis/Wilcoxon comparison tests.

† p < 0,05.

– It was not possible to calculate standard deviation with just one observation.

The number of consultations also differs significantly according to mothers’ education and color (p < 0.001). It is observed that women with 8 to 11 years of education and 12 years or more had a higher average of prenatal consultations. As for color, the most frequent category was white. This group, together with brown, yellow and indigenous people had means and medians of number of consultations greater than nine, unlike those of the black or ignored color (Table 1).

Still, regarding pregnancy type, it was possible to observe that the majority had a single pregnancy. It is noteworthy that mothers whose pregnancy type was multiple had higher averages of prenatal consultations, such differences being considered significant (p = 0.001) (Table 1).

Table 2 allows us to verify that newborns’ mothers were, on average, 31.50 years old, and that this age has a weak and positive correlation with the number of prenatal consultations, indicating that the higher the maternal age, the greater tends to be the number of prenatal consultations performed.

**Table 2.** Number of prenatal consultations performed by mothers of children stratified as intermediate risk according to maternal age, number of previous pregnancies, number of cesarean deliveries, number of normal births, number of live children, number of dead children and month of pregnancy that started prenatal care – Maringá, Paraná, Brazil, 2019.

Variable	Descriptive measures				Prenatal consultations	
	N	Mean	Standard deviation	Median	r_s_*	p^†^
Mother’s age	1219	31.50	6.83	32	0.144	<0.001^‡^
Number of previous pregnancies	1217	2.01	1.27	2	–0.010	0.723
Number of cesarean deliveries	1217	0.69	0.89	0	0.016	0.584
Number of normal births	1216	0.50	0.93	0	–0.091	0.001^‡^
Number of living children	1217	1.07	1.07	1	–0.097	0.001^‡^
Number of dead children	1217	0.96	0.76	1	0.129	<0.001^‡^
Month of pregnancy in which prenatal care started	1212	2.14	3.03	2	–0.285	<0.001^‡^

Source: Municipal Health Office of Maringá, Paraná, Brazil, 2019. * The coefficient indicates the correlation direction, inverse (negative) or direct (positive), while the value indicates the correlation force. ^†^ p value calculated from Spearman’s comparison test. ^‡^ p value < 0.05.

There is not enough sample evidence that the number of previous pregnancies and cesarean deliveries is significantly correlated with the number of prenatal consultations, unlike the number of normal births, number of live births and number of stillbirths. It is noteworthy that all correlations were weak and, for the number of dead children, the correlation was positive, while for the other variables it was negative (Table 2).

With regard to perinatal outcomes, it is observed in Table 3 that 99.59% of births were performed in the hospital, 99.59% were assisted by a medical professional and 89.75% did not have induced labor, and that the number of prenatal consultations did not presents significant differences with such characteristics.

**Table 3.** Number of prenatal consultations performed by mothers of children stratified as intermediate risk according birthplace, professional who assisted birth, delivery type, if labor was induced, if the cesarean was performed before labor, newborn presentation, method used to estimate the gestational age, newborn sex and presence of anomaly – Maringá, Paraná, Brazil, 2019.

Variable	Frequency		Prenatal consultations			p*
	N	%	Mean	Standard deviation	Median	
Birthplace						0.209
Hospital	1214	99.59%	9.51	3.60	9	
Home	4	0.33%	9.00	2.16	8.5	
Other health facilities	1	0.08%	38.00	–	38	
Birth was assisted by whom?						0.287
Doctor	1214	99.59%	9.54	3.68	9	
Nurse/midwife	5	0.41%	7.20	4.44	8	
Delivery type						<0.001^†^
Cesarean section	949	77.85%	9.84	3.67	10	
Normal	270	22.15%	8.42	3.52	8	
Was labor induced?						0.248
No	1094	89.75%	9.51	3.73	9	
Yes	124	10.17%	9.65	3.32	9	
Ignored	1	0.08%	15.00	–	15	
Did cesarean occur before labor started?						<0.001^†^
No	218	17.88%	9.47	3.69	10	
Yes	695	57.01%	10.00	3.65	10	
Not applicable	34	2.79%	8.88	3.84	8	
Ignored	272	22.31%	8.45	3.53	8	
Newborn presentation type						0.760
Cephalic	1138	93.36%	9.49	3.58	9	
Pelvic or podalic	70	5.74%	9.93	5.14	9	
Transverse	3	0.25%	10.00	1.00	10	
Ignored	8	0.66%	11.13	4.32	9.5	
Method employed						0.024^†^
Physical examination	1	0.08%	8.00	–	8	
Alternative method	1115	91.47%	9.39	3.50	9	
Ignored	103	8.45%	11.01	5.10	10	
Sex						0.989
Male	639	52.42%	9.56	3.82	9	
Female	580	47.58%	9.50	3.54	9	
Anomaly identified						0.921
No	1211	99.34%	9.53	3.69	9	
Yes	8	0.66%	9.50	3.30	9	

Source: Municipal Health Office of Maringá, Paraná, Brazil, 2019. * p value calculated from Kruskal-Wallis/Wilcoxon comparison tests. ^†^ p value < 0.05. – It was not possible to calculate the standard deviation with just one observation.

On the other hand, significance was observed in relation to delivery type and occurrence of cesarean section before labor began. Mothers whose delivery was cesarean had, in general, a greater number of prenatal consultations in relation to normal delivery, as did mothers who had a cesarean section before the beginning of labor (Table 3).

It was found that most of newborns had a cephalic presentation, had no abnormalities and just over half were male. Only the method for estimating gestational age, which usually uses ultrasound exams, showed significance in relation to the difference in the number of prenatal consultations (p = 0.024).

Finally, Table 4 shows that only Apgar at the 5^th^ minute significantly correlates with the number of prenatal consultations, positively. Newborns had an average of 37.91 weeks of gestation, with no significant correlation with the number of prenatal consultations, unlike birth weight (p = 0.012).

**Table 4.** Number of prenatal visits performed by mothers of children stratified as intermediate risk according to Apgar at the 1^st^ and 5^th^ minutes, Robson group code and number of weeks of gestation – Maringá, Paraná, Brazil, 2019.

Variable	Descriptive measures						Prenatal consultations	
	N	Mean	Standard deviation	Median	Minimum – Maximum	IQR	rSrS*	p^†^
Apgar at the 1^st^ minute	1218	8.34	1.33	9	(0 – 10)	(8 – 9)	0.042	0.146
Apgar at the 5^th^ minute	1218	9.38	0.79	9.5	(3 – 10)	(9 – 10)	0.114	<0.001^‡^
Robson group code	1219	4.70	2.60	5	(1 – 11)	(3 – 5)	–0.027	0.347
Number of gestation weeks	1216	37.91	2.24	38	(23 – 44)	(37 – 39)	0.036	0.207
Weight	1219	3151.84	614.73	3205	(470 – 4976)	(2850 – 3564)	0.072	0.012^‡^

Source: Municipal Health Office of Maringá, Paraná, Brazil, 2019. The coefficient indicates the correlation direction, inverse (negative) or direct (positive), while the value indicates the correlation force. ^†^ p value calculated from Spearman’s comparison test. ^‡^ p value < 0.05. IQR: interquartile range.

## DISCUSSION

The present study analyzed the number of prenatal consultations as a function of maternal characteristics, in order to observe to what extent these factors influence the follow-up process. Additionally, we tried to analyze the repercussions of this on perinatal outcomes. In this perspective, it was found that women with a partner, higher level of education, self-declared white and aged over 30 years were more frequent prenatal care, whereas the newborns of mothers with the ideal number of consultations were born with better health conditions.

It is known that adequate prenatal care is associated with a reduction in maternal and child deaths. The results also showed that most mothers of newborns had at least nine prenatal consultations. In general, studies have shown that pregnant women are attending prenatal care; however, it is believed that the problem may be related to the quality of care that is being offered by services^(3,11–12)^.

Married women or in a stable relationship were the ones who most attended the consultations, in line with the findings of several studies, which indicate that pregnant women with the support of their husband/partner, with better economic and psychological conditions during pregnancy, are more frequent in prenatal monitoring^(13–14)^.

Maternal education is also an important element in child mortality. Pregnant women with more education years had the highest average number of prenatal consultations. This data allows us to infer that the greater the level of education, the greater the understanding of the importance of prenatal care for mother and baby. It is also worth mentioning that low education, isolated from other factors, increases the chances of neonatal death by 25%, according to a study that analyzed maternal education and age related to neonatal mortality^(15–16)^.

With regard to color, the survey registered a greater number of consultations among white pregnant women, representing more than half of all other skin color categories. On the other hand, black pregnant women and those of ignored color were those who registered the lowest number of prenatal consultations. A similar study, which aimed to classify and estimate risk-associated factors during pregnancy, identified black as the most frequent color among intermediate-risk pregnant women in the same study municipality^(13)^.

At the national level, research carried out in the north of Bahia identified black or brown pregnant women with inadequate prenatal care, absence of a companion in the maternity ward, pilgrimage through maternity hospitals to receive specialized care and less instruction on the process of pregnancy, birth and in-hospital care^(17)^.

These data confirm the patterns of ethnic inequalities between Brazilian regions, which have been described in several investigations in Brazil, leading us to reflect on whether public policies aimed at maternal and child health have the same reach for all population strata. It is important to emphasize that socioeconomic and ethnic factors can express the vulnerability of some groups of women and children^(18–19)^. This aspect reinforces the need to comply with criteria based on such factors, in order to seek to ensure special and more personalized attention by health services to this portion of the population.

As well as education and color, maternal age can significantly influence infant mortality rates, especially in the neonatal component^(15)^. The study showed that, the older the pregnant woman is, the greater the number of consultations performed by her.

The relationship between maternal age and pregnancy complications is also reported by other authors, who attribute to extremes of age, a higher incidence of obstetric problems and a higher frequency of hospitalizations^(14)^. In this context, it is clear that teenage pregnancy represents a public health problem faced in most emerging countries. These teenagers are often exposed to unfavorable socioeconomic situations, such as low education, unpaid work, lack of opportunities and professional qualification, in addition to the absence of family support, making them vulnerable, which can contribute to the lack of behavior during prenatal care^(16)^.

Maternal age above 35 is also highlighted, as pregnant women with this profile tend to be multiparous, which contributes to a higher risk of complications during pregnancy. Furthermore, it is known that, as a woman ages, more risk factors are added to the pregnancy^(13)^. Research carried out in the state of Rio de Janeiro analyzed neonatal deaths over a period of seven years and found that children of adolescents and women aged 35 years or over had a greater chance of mortality, when compared to children of mothers aged between 20 and 34 years old^(15)^.

Regarding the types of births analyzed in this study, it is observed that almost 78% of them were cesarean deliveries and that, in more than half of the deliveries, cesarean section occurred even before labor started. These data are in line with the findings of other studies that already show Brazil as the second country in the world that performs more cesarean deliveries, only behind the Dominican Republic^(20–21)^.

Research conducted in Brazil and in the states analyzed the main causes of death in childhood in 1990 and 2015, identifying that, despite the falls suffered in the period, prematurity was the main cause of death in both years, a fact that may be related to the high rates of cesarean sections performed in the country^(22)^.

International, national organizations and managers warn of the high rates of cesarean sections in the country, which represent 40% in the public health sector and 84% in the private network^(23)^. Women with better socioeconomic status and without risk factors for vaginal birth make up the majority in cesarean delivery statistics^(3)^.

Also noteworthy are Apgar scores at the 1^st^ and 5^th^ minutes. The latter was positively related to the number of prenatal consultations performed. It is a consensus that monitoring pregnant women improves the conditions of delivery and birth for the binomial^(2)^.

A study carried out in the national scenario, which analyzed the health conditions of newborns in relation to the number of consultations carried out by the mothers, showed that newborns with Apgar at the 1_st_ minute ranging from 8 to 10 were pregnant women who had more than seven consultations. For those who had one to three consultations or none, Apgar ranged from 0 to 2. Regarding the Apgar assessed at the 5^th^ minute, it remained from 8 to 10 for those who attended more than seven appointments, and ranged from 3 to 5 for those who attended between six to four appointments. Moreover, for those who did not undergo follow-up, this index was from 0 to 2, showing the relevance of prenatal care in newborns’ health^(24)^.

With regard to gestational age, the same study showed that pregnant women who attended seven appointments or more had their babies at term, i.e., between 37 and 42 weeks. On the other hand, for those who attended from four to six consultations, the deliveries took place within the period of 28 to 31 weeks. For those who only had one to three consultations, the children were born between 22 and 27 weeks, and for pregnant women who did not attend prenatal care, they gave birth before the 22^nd^ week of gestation^(24)^.

The study results also reveal the positive relationship between prenatal consultations and birth weight, corroborating other authors who attributed the ideal number of consultations, adequate birth weight, as well as better health conditions, lower frequency of hospitalizations and complications arising from birth^(12,24)^.

With regard to preventable causes of death in children under one year of age, a study identified birth weight, along with prematurity, as the main factors that interfere with child health. However, both conditions are preventable by quality prenatal care, which refer to the adequate number of consultations by pregnant women, emphasizing the importance that these are offered by professionals trained to work in neonatal care^(11)^.

In this sense, it is expected that the study will contribute to the nursing practice of professionals working in maternal and child health services, recognizing the importance of observing the criteria for risk stratification of pregnant women, in order to promote the implementation of qualified and resolute care, with a view to improving the perinatal results of newborns and complying with the guidelines proposed by the current programs. The present study, by focusing its attention on the intermediate risk of a state maternal and child care program, encourages professionals and trainers to reflect on the importance of considering epidemiological and sociodemographic aspects as determinants of health behavior, and as essential elements for the systematic and continuous assessment of public policies so that they are in fact consistent with the population’s realities and needs.

With the transition from PRMP to Maternal and Child Care Line, recently announced by the *Secretaria do Estado da Saúde do Paraná* (SESA – Paraná State Health Department), future research will be needed in order to assess the impacts of changes on the health of this clientele, especially those classified as intermediate risk^(25)^.

Finally, this study has limitations regarding the use of secondary data obtained from the CLB, subject to incomplete information. However, the Live Birth Information System is still a fundamental source for conducting epidemiological studies on maternal and child vital statistics, with national and regional coverage.

## CONCLUSION

The results presented in the study highlight the importance of prenatal care, through regular consultations at maternal and child health services, as well as the positive impact of these on the perinatal outcomes of newborns.

Pregnant women with higher education, white, with a partner and aged 30 years or more had adequate prenatal care from a numerical point of view, with seven appointments or more. As a result, their newborns had better perinatal outcomes, reflected in Apgar scores and birth weight.

In this context, the role of nurses is of paramount importance as leaders of initiatives to integrate actions in the multidisciplinary team, whether at the level of Primary Health Care, or in hospital and specialized care. It is essential raise awareness of these professionals about the importance of knowing and considering the maternal characteristics that represent risk factors for the mother-child dyad, performing risk stratification, and offering specialized care, in order to contribute to healthy births and reduction resumption in infant mortality.

## Financial support

This study was financially supported by Coordenação de Aperfeiçoamento de Pessoal de Nível Superior – Brazil (CAPES) – Financing code 001. 

## References

[1] Unicef (2020). Levels and Trends in Child Mortality.

[2] Maia LTS, Souza WV, Mendes ACG (2020). Individual and contextual determinants of infant mortality in
Brazilian state capitals: a multilevel approach. Cad Saude Publica..

[3] Leal MC, Szwarcwald CL, Almeida PVB, Aquino EML, Barreto ML, Barros F (2018). Reproductive, maternal, neonatal and child health in the 30 years
since the creation of the Unified Health System (SUS). Cien Saude Colet..

[4] World Health Organization (2020). Levels & Trends in Child Mortality. Report 2020 Estimates developed
by the UN Inter-agency Group for Child Mortality Estimation.

[5] Ayres JR, Castellanos MEP, Baptista TWF (2018). Interview with José Ricardo Ayres. Saúde e Sociedade..

[6] Secretaria de Estado da Saúde do Paraná (2018). Linha Guia Rede Mãe Paranaense.

[7] Soares JHR, Caldeira S, Zani AV, Ferrari RAP, Silva RMM, Tacla MTGM (2017). The Rede Mãe Paranaense Program from the perspective of primary
health care nurses. Revista Eletrônica Acervo Saúde.

[8] Rocha RRM, França AFO, Zilly A, Caldeira S, Machineski GG, Silva RMM (2018). Nurses’ knowledge and perception in the maternal and child health
network of Paraná. Cien Saude Colet..

[9] Elm EV, Altman DG, Egger M, Pocock SJ, Gøtzsche PC, Vandenbroucke JP (2007). The Strengthening the Reporting of Observational Studies in
Epidemiology (STROBE) Statement: guidelines for reporting observational
studies. The BMJ..

[10] Instituto Brasileiro de Geografia e Estatística (2017). População.

[11] Lisboa L, Abreu DMX, Lana AMQ, França EB (2015). Infant mortality: leading avoidable causes in the central region
of Minas Gerais, Brazil, 1999-2011. Epidemiol Serv Saude..

[12] Silva AC, Migoto MT, Souza SJP, Tomin LL (2019). Indicadores de mortalidade perinatal, infantil e materna regional
de saúde do Estado do Paraná. Revista Gestão & Saúde.

[13] Novaes ES, Melo MC, Ferracioli PLRV, Oliveira RR, Mathias TAF (2018). Gestational risk and associated factors in women cared by the
public health network. Ciência, Cuidado e Saúde..

[14] Gomes FCS, Aragão FBA, Serra LLL, Chein MBC, Santos JPF, Santos LMR (2020). Relationship between stress and self-esteem of pregnant women
during prenatal care. Medicina..

[15] Fonseca SC, Flores PVG, Camargo KR, Pinheiro RS, Coeli CM (2017). Maternal education and age: inequalities in neonatal
death. Rev Saude Publica..

[16] Prezotto KH, Oliveira LR, Oliveira RR, Melo EC, Scholze AR, Fernandes CAM (2019). Child mortality: trend and changes after the implantation of the
rede mãe paranaense program. Enfermería Global..

[17] Oliveira ADF, Campelo MJA (2020). Prenatal care in the rural area, in northern Bahia -BA: Pregnant
women profile attended at the nursing consultations. Brazilian Journal of health Review..

[18] Caldas ADR, Santos RV, Borges GM, Valente JG, Portela MC, Marinho GL (2017). Infant mortality according to color or race based on the 2010
Population Census and national health information systems in
Brazil. Cad Saude Publica..

[19] Sanine PR, Venancio SI, Silva FLG, Aratani N, Moita MLG, Tanaka OY (2019). Prenatal care in high-risk pregnancies and associated factors in
the city of São Paulo, Brazil. Cad Saude Publica..

[20] Federação Brasileira das Associações de Ginecologia e
Obstetrícia (c2022). Alta taxa de cesáreas no país é tema de audiência pública.

[21] Boerma T, Ronsmans C, Melesse DY, Barros AJD, Barros FC, Juan L (2018). Global epidemiology of use of and disparities in caesarean
sections. The Lancet..

[22] França EB, Lansky S, Rego MAS, Malta DC, França JS, Teixeira R (2017). Leading causes of child mortality in Brazil, in 1990 and 2015:
estimates from the Global Burden of Disease study. Rev Bras Epidemiol..

[23] Unicef (2017). Quem Espera, Espera.

[24] Pereira RF (2019). Relação das consultas de pré-natal e as condições de saúde dos
recém-nascidos no Brasil, 2013-2017.

[25] Governo do Estado do Paraná, Secretaria da Saúde Linha de Atenção Materno Infantil.

